# Influenza Incidence and Vaccine Effectiveness During the Southern Hemisphere Influenza Season — Chile, 2022

**DOI:** 10.15585/mmwr.mm7143a1

**Published:** 2022-10-28

**Authors:** María Fernanda Olivares Barraza, Rodrigo A Fasce, Francisco Nogareda, Perrine Marcenac, Natalia Vergara Mallegas, Patricia Bustos Alister, Sergio Loayza, Anna N. Chard, Carmen Sofia Arriola, Paula Couto, Christian García Calavaro, Angel Rodriguez, David E. Wentworth, Cristóbal Cuadrado, Eduardo Azziz-Baumgartner

**Affiliations:** ^1^Ministry of Health, Santiago, Chile; ^2^Virology Department, Public Health Institute of Chile, Santiago, Chile; ^3^Pan American Health Organization, Washington, DC; ^4^Influenza Division, National Center for Immunization and Respiratory Diseases, CDC.

The COVID-19 pandemic has affected influenza virus transmission, with historically low activity, atypical timing, or altered duration of influenza seasons during 2020–22 (*1*,*2*). Community mitigation measures implemented since 2020, including physical distancing and face mask use, have, in part, been credited for low influenza detections globally during the pandemic, compared with those during prepandemic seasons (*1*). Reduced population exposure to natural influenza infections during 2020–21 and relaxed community mitigation measures after introduction of COVID-19 vaccines could increase the possibility of severe influenza epidemics. Partners in Chile and the United States assessed Southern Hemisphere influenza activity and estimated age-group–specific rates of influenza-attributable hospitalizations and vaccine effectiveness (VE) in Chile in 2022. Chile’s most recent influenza season began in January 2022, which was earlier than during prepandemic seasons and was associated predominantly with influenza A(H3N2) virus, clade 3C.2a1b.2a.2. The cumulative incidence of influenza-attributable pneumonia and influenza (P&I) hospitalizations was 5.1 per 100,000 person-years during 2022, which was higher than that during 2020–21 but lower than incidence during the 2017–19 influenza seasons. Adjusted VE against influenza A(H3N2)-associated hospitalization was 49%. These findings indicate that influenza activity continues to be disrupted after emergence of SARS-CoV-2 in 2020. Northern Hemisphere countries might benefit from preparing for an atypical influenza season, which could include early influenza activity with potentially severe disease during the 2022–23 season, especially in the absence of prevention measures, including vaccination. Health authorities should encourage all eligible persons to seek influenza vaccination and take precautions to reduce transmission of influenza (e.g., avoiding close contact with persons who are ill).

Influenza incidence was estimated using Chile’s Ministry of Health (Ministerio de Salud [MINSAL]) Department of Statistics and Health Information hospital discharge data and viral surveillance data from the National Influenza Centre (NIC). Chile registers all public and private hospital patient discharge diagnoses in a central data set. A subset of respiratory specimens from these patients was tested using CDC reverse transcription–polymerase chain reaction (RT-PCR) protocols^†^ for influenza virus during routine clinical care or as part of national respiratory virus surveillance. The epidemic threshold used to delineate the influenza season was defined as the mean of weekly percentage of positive specimens tested through NIC during 2017–19. The start of the influenza season for each calendar year during 2017–19 and in 2022 was defined as the epidemiologic week during which the percentage of influenza-positive specimens had exceeded the historical epidemic threshold for ≥3 weeks. Previously described methods (*3*) were used to estimate cumulative incidence of influenza hospitalization. Only certain *International Classification of Diseases, Tenth Revision* (ICD-10) P&I discharge diagnosis codes (J09–18) were considered to be attributable to influenza viruses because providers typically make these diagnoses in the absence of laboratory testing. To attribute P&I diagnoses to influenza, the percentage of patients with severe acute respiratory infections (SARIs) enrolled from nine sentinel sites^§^ with influenza-positive specimens tested at NIC was applied to untested patients with a P&I diagnosis. The percentage of SARI patients with an influenza-positive specimen was calculated for each month and for each age group (<5, 5–18, 19–64, and ≥65 years). A similar proportion of persons with P&I diagnoses were assumed to have a positive influenza test result (Supplementary Table; https://stacks.cdc.gov/view/cdc/121863). To minimize misclassification, only cases for which P&I was the principal diagnosis associated with hospitalization were included in calculations estimating the influenza-attributable proportion of P&I cases. The age group–specific proportion was calculated by age group and by month, totaled for each age group, divided by the number of persons in that age group for that year, and then multiplied by 100,000 to derive incidence per 100,000 person-years.

In 2022, Chile used Abbott INFLUVAC, a Southern Hemisphere, trivalent egg-based influenza vaccine formulation containing antigens from an A/Victoria/2570/2019 (H1N1)pdm09–like virus, A/Darwin/9/2021 (H3N2)–like virus, and B/Austria/1359417/2021 (B/Victoria lineage)–like virus (*4*). SARI sentinel data submitted to the Pan American Health Organization’s Network for the Evaluation of Vaccine Effectiveness in Latin America and the Caribbean — influenza (REVELAC-i ) were used to estimate this vaccine’s effectiveness in preventing influenza hospitalizations using previously described methods (*5*). REVELAC-i used a test-negative, case-control design to determine the likelihood that a hospitalized patient with severe respiratory infection and a positive influenza test result (case-patient) had been previously vaccinated against influenza compared with the odds that a patient hospitalized with a similar illness, but with a negative influenza test result (control-patient), had been vaccinated. Patients with positive SARS-CoV-2 RT-PCR test results were excluded from the control group (*6*). VE estimates were calculated as 1 − odds ratio x 100 and adjusted for age, month of symptom onset, and preexisting conditions. This report was reviewed by MINSAL and conducted consistent with relevant laws.^¶^ This activity was reviewed by CDC and was conducted consistent with applicable federal law and CDC policy.**

During 2022, Chile’s NIC tested 59,392 respiratory specimens through its national laboratory network, 3,140 (5.3%) of which were positive for SARS-CoV-2, and 4,070 (6.9%) of which were positive for influenza. Among influenza-positive specimens, 2,204 (54%) were typed, and all but one (2,203; >99.9%) were influenza A(H3N2) virus; the remaining one specimen was an influenza A(H1N1) virus. During 2017, 2018, and 2019, the influenza epidemic threshold was 6.2%, and the start of influenza season occurred during weeks 18, 21, and 17, respectively, corresponding to an influenza season beginning during April–May. In contrast, in 2022, the percentage of influenza-positive specimens first surpassed and remained above this epidemic threshold during weeks 1–6 (January–February), was below the epidemic threshold during weeks 7–17, and then surpassed it again starting in week 18 (May); peak activity was during week 24 (June) (Figure). All 280 (12.7%) influenza virus specimens sequenced by next-generation sequencing were influenza A(H3N2), clade 3C.2a1b.2a.2.

**FIGURE:**
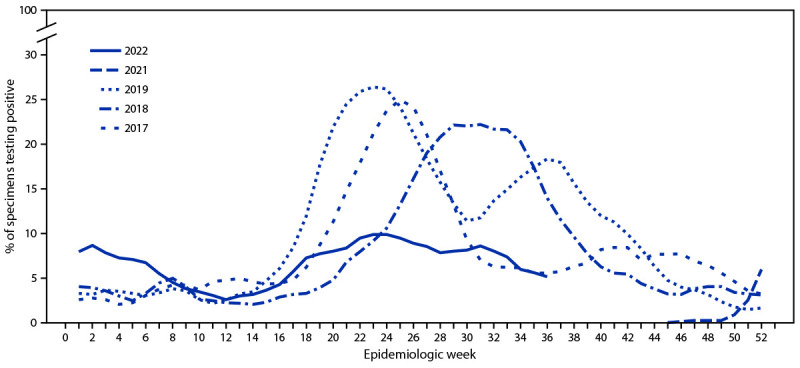
Percentage of respiratory specimens testing positive for influenza virus,* by epidemiologic week — National Influenza Centre, Chile, 2017–19 and 2021–22^†^ * Data lines represent right-aligned, 3-week moving averages of percentage of specimens testing positive for influenza virus. ^†^ 2022 data as of epidemiologic week 36.

During January–August 2022, a total of 17,752 (0.1%) persons in Chile’s population (19,828,563) were hospitalized for treatment of P&I; among these patients, 4,911 (27.7%) were aged <5 years, 929 (5.2%) were aged 5–18 years, 3,342 (18.8%) were aged 19–64 years, and 8,570 (48.3%) were aged ≥65 years (Table). A total of 6,025 SARI patients were enrolled at nine Chile sentinel surveillance sites in 2022. Among these, respiratory specimens from 5,731 (95.1%) patients were tested by RT-PCR; 301 (5.3%) of these were positive for influenza virus.

**TABLE:** Influenza-attributable hospitalizations by age group — Chile, epidemiologic weeks 1–32, 2022

Age group, yrs	No. of patients (column %)		No. (%)^§^ of SARI patients with respiratory specimens tested^†^	No. (%)^¶^ of influenza-positive specimens^†^	No. of P&I diagnoses attributable to influenza**	Population^††^	Incidence^§§^ (95% CI)
	With P&I clinical influenza discharge diagnosis*	With SARI, enrolled at sentinel sites^†^					
**Total**	**17,752 (100.0)**	**6,025 (100.0)**	**5,731 (95.1)**	**301 (5.3)**	**1,002**	**19,828,563**	**5.1 (4.8**–**5.4)**
<5	4,911 (27.7)	1,927 (32.0)	1,880 (97.6)	49 (2.6)	132	1,177,286	11.2 (9.4–13.3)
5–18	929 (5.2)	424 (7.0)	401 (94.6)	31 (7.7)	79	3,542,159	2.2 (1.8–2.8)
19–64	3,342 (18.8)	1,315 (21.8)	1,217 (92.5)	99 (8.1)	302	12,548,497	2.4 (2.2–2.7)
≥65	8,570 (48.3)	2,359 (39.2)	2,233 (94.7)	122 (5.5)	521	2,560,621	20.3 (18.7–22.2)

**Abbreviations:** P&I = pneumonia and influenza; SARI = severe acute respiratory infection.

* National-level hospital discharge data from Department of Statistics and Health Information, Chile’s Ministry of Health (Ministerio de Salud), with *International Classification of Diseases, Tenth Revision* hospital discharge codes J09–J18. https://deis.minsal.cl

^†^ SARI sentinel surveillance implemented in nine tertiary care hospitals in northern, central, and southern Chile (Hospital de Antofagasta, Antofagasta; Hospital de Magallanes, Punta Arenas; Hospital de Puerto Montt, Puerto Montt; Hospital Ernesto Torres Galdámez, Iquique; Hospital Guillermo Gran Benavente, Concepción; Hospital Gustavo Fricke, Viña del Mar; Hospital Hernán Enriquez Aravena, Temuco; Hospital Militar, Santiago; and Hospital San Juan de Dios, Santiago).

^§^ Percentage of enrolled SARI patients.

^¶^ Percentage of tested specimens from SARI patients.

** Age-specific numbers in this column do not sum to total because of rounding when calculating influenza-attributable P&I diagnoses from P&I hospitalizations and SARI data.

^††^ Population projections and estimates calculated based on 2017 census data from the National Institute of Statistics, Santiago, Chile.

^§§^ Cases per 100,000 person-years; incidence estimated using World Health Organization-recommended methods. https://apps.who.int/iris/handle/10665/178801

Overall, 1,002 (5.6%) of 17,752 P&I hospitalizations were attributable to influenza; among these, 132 (12.8%) were among persons aged <5 years, 79 (7.6%) were among persons aged 5–18 years, 302 (29.2%) were among adults aged 19–64 years, and 521 (50.4%) were among adults aged ≥65 years.^††^ The cumulative incidence of influenza-attributable P&I hospitalizations during weeks 1–32 was 5.1 per 100,000 person-years during 2022. The highest incidence (20.3 per 100,000 person-years) was among adults aged ≥65 years. Among persons aged <5 years, 5–18 years, and 19–64 years, incidences were 11.2, 2.2, and 2.4, respectively. Incidence of influenza-attributable P&I hospitalizations during 2022 was substantially higher than that in 2021 (0.01) and 2020 (0.6), but substantially lower than that in 2017 (28.7), 2018 (23.0), and 2019 (30.4).

Among persons prioritized to receive influenza vaccination in Chile during 2022 (adults aged ≥65 years, persons aged 11–64 years with chronic conditions, pregnant women, infants and children aged 6 months–10 years, and certain other persons^§§^) who accounted for 41% of the total population, 92.5% were vaccinated. Although 2022 Southern Hemisphere formulation vaccines were not available before the first unseasonal influenza wave during weeks 1–6, approximately 88% of vaccinated persons received their vaccine before the peak of 2022 influenza activity in week 24.

Sentinel surveillance data submitted to REVELAC-i used to estimate VE for Chile included 717 test-negative control-patients and 175 case-patients. Among 175 case-patients, 118 (67%) received a positive test result for A(H3N2) virus, and one received a positive test result for A(H1N1)pdm09 virus. The crude and adjusted estimates of VE against influenza A(H3N2)-associated hospitalization were 46% (95% CI = 17%–65%) and 49% (95% CI = 23%–67%), respectively.

## Discussion

The influenza epidemic in Chile during 2022 began months earlier than in a typical influenza season (*7*) and resulted in 1,002 influenza-associated P&I hospitalizations. Whereas 2022 incidence of influenza-associated hospitalization was four to six times lower than during 2017–19 pre–COVID-19 pandemic seasons, it was much higher than incidence in 2020–21 when influenza virus detection in Chile was low. Chile’s influenza activity in 2022, including an early unseasonal start and A(H3N2) virus predominance, was consistent with trends in other countries in the Southern Hemisphere in 2022, including Australia, Argentina, and Peru; the start of South Africa’s influenza seasons was consistent with prepandemic seasons and was characterized by an initial predominance of A(H1N1)pdm09, followed by B/Victoria viruses.^¶¶^

To reduce influenza-associated morbidity, the government of Chile vaccinated >90% of persons prioritized for vaccination free of charge. Although these vaccines only became available after the first influenza wave (weeks 1–6), Chilean health authorities successfully vaccinated 88% of the target population before peak influenza activity in week 24. Influenza vaccines were 49% effective at preventing hospitalizations during this predominantly A(H3N2) clade 3C.2a1b.2a.2 season. The Northern Hemisphere 2022–23 influenza vaccine formulation contains the same A(H3N2) clade and antigen (3C.2a1b.2a.2 and A/Darwin/9/2021, respectively) used in the 2022 Southern Hemisphere vaccine; if the A(H3N2) clade 3C.2a1b.2a.2 also predominates during 2022–23 Northern Hemisphere influenza season, this Northern Hemisphere formulation might be similarly effective at preventing severe influenza illnesses. Like certain Southern Hemisphere jurisdictions, Chile identified a limited number of influenza B virus cases, none of which was subtyped as B/Yamagata. The global absence of B/Yamagata might indicate that this subtype has become rare (*8*); however, continued surveillance is needed to learn whether it will reemerge in future seasons.

The findings in this report are subject to at least three limitations. First, the dominant circulating subtype was determined based on the typing of 54% (2,204 of 4,070) of respiratory specimens that tested positive for influenza virus, and the dominant clade was determined from sequencing 13% of specimens (280 of 2,203); thus, it is possible that other virus types and clades were not identified. Second, VE estimates are based on a limited number of hospitalized case-patients and control-patients from nine hospitals, and unmeasured confounding, including confounding associated with hospitalization or vaccination, might be present in this data set. Finally, testing and hospitalization data could have been affected by changes in health care–seeking behavior because of the COVID-19 pandemic which was not assessed in these analyses.

These data from Chile’s 2022 influenza season indicate that influenza activity in the Southern Hemisphere was atypical, likely because of continued effects of emergence of SARS-CoV-2 in 2020. Strict observance of community mitigation measures (*9*) and high influenza vaccination coverage likely mitigated influenza incidence in Chile during the 2022 season. Northern Hemisphere countries could benefit from preparing for an atypical season, which could include early influenza activity with potentially severe disease for the 2022–23 season, especially in the absence of prevention measures, including vaccination. Health officials should encourage communities to protect themselves by seeking influenza vaccination in accordance with CDC recommendations and taking precautions to reduce transmission of influenza, including avoiding close contact with persons who are ill (*10*).

**Summary**

**What is already known about this topic?**

Influenza transmission has changed during the COVID-19 pandemic.

**What is added by this report?**

In 2022, influenza A(H3N2) virus, clade 3C.2a1b.2a.2, circulated in Chile months earlier than during prepandemic influenza seasons and was associated with 1,002 hospitalizations. Influenza vaccination reduced risk for A(H3N2) virus hospitalization by 49%.

**What are the implications for public health practice?**

Like certain Southern Hemisphere countries during the 2022 influenza season, Northern Hemisphere countries might face influenza activity with atypical timing and intensity during the 2022–23 season. Health authorities should encourage all eligible persons to seek influenza vaccination and take precautions to reduce transmission of influenza (e.g., avoiding close contact with persons who are ill).

## Footnotes

* These authors contributed equally to this report.

†
https://www.cdc.gov/coronavirus/2019-ncov/lab/multiplex.html

§ SARI sentinel surveillance implemented in nine tertiary care hospitals in northern, central, and southern Chile (Hospital de Antofagasta, Antofagasta; Hospital de Magallanes, Punta Arenas; Hospital de Puerto Montt, Puerto Montt; Hospital Ernesto Torres Galdámez, Iquique; Hospital Guillermo Gran Benavente, Concepción; Hospital Gustavo Fricke, Viña del Mar; Hospital Hernán Enriquez Aravena, Temuco; Hospital Militar, Santiago; and Hospital San Juan de Dios, Santiago).

¶ This report does not violate Chilean Law No. 20.584 that “Regulates the rights and duties that people have in relation to actions related to their health care,” because MINSAL obtains all the information in compliance with its role according to previous laws DFL No. 1/2005 of the MINSAL and Law No. 19.628 on sensitive data.

** 45 C.F.R. part 46.102(l)(2), 21 C.F.R. part 56; 42 U.S.C. Sect. 241(d); 5 U.S.C. Sect. 552a; 44 U.S.C. Sect. 3501 et seq.

†† Age-specific numbers do not sum to total because of rounding when calculating influenza-attributable P&I diagnoses from P&I hospitalizations and SARI data.

§§ Influenza vaccination priority groups in Chile include health care personnel, persons aged ≥65 years, persons aged 11–64 years with chronic conditions, pregnant women, infants and children aged 6 months–10 years, premature infants with specific pathologies, educations workers, and agricultural workers who work with poultry or swine.

¶¶ https://www.who.int/tools/flunet (Accessed September 22, 2022).
